# Muscle synergies inherent in simulated hypogravity running reveal flexible but not unconstrained locomotor control

**DOI:** 10.1038/s41598-023-50076-6

**Published:** 2024-02-01

**Authors:** Camille Fazzari, Robin Macchi, Yoko Kunimasa, Camélia Ressam, Rémy Casanova, Pascale Chavet, Caroline Nicol

**Affiliations:** 1 Aix-Marseille Univ, CNRS, ISM, Marseille, France; 2 French Institute of Sport (INSEP), Laboratory Sport, Expertise and Performance (EA 7370), Paris, France; 3 Niigata University, Niigata, Japan; 4 NeuroSpin, UMR CEA/CNRS 9027, Paris-Saclay University, Gif-sur-Yvette, France

**Keywords:** Motor control, Neurophysiology

## Abstract

With human space exploration back in the spotlight, recent studies have investigated the neuromuscular adjustments to simulated hypogravity running. They have examined the activity of individual muscles, whereas the central nervous system may rather activate groups of functionally related muscles, known as muscle synergies. To understand how locomotor control adjusts to simulated hypogravity, we examined the temporal (motor primitives) and spatial (motor modules) components of muscle synergies in participants running sequentially at 100%, 60%, and 100% body weight on a treadmill. Our results highlighted the paradoxical nature of simulated hypogravity running: The reduced mechanical constraints allowed for a more flexible locomotor control, which correlated with the degree of spatiotemporal adjustments. Yet, the increased temporal (shortened stance phase) and sensory (deteriorated proprioceptive feedback) constraints required wider motor primitives and a higher contribution of the hamstring muscles during the stance phase. These results are a first step towards improving astronaut training protocols.

## Introduction

Human running partly results from musculoskeletal specializations that arose 2 million years ago^1^. On Earth, it uses a mass-spring mechanism: Muscle–tendon units store elastic energy during the initial braking phase of the running cycle, and release it during the subsequent push-off phase^2,3^. This occurs because the musculoskeletal system is periodically subjected to impact and stretching forces, as a direct result of Earth’s gravity (1 g ~ 9.81 m.s^−2^). At a time when human missions to the Moon (0.16 g) and Mars (0.38 g) are being planned, it is critical to understand how locomotor control adjusts to hypogravity (0 < g < 1).

**Figure 1:**
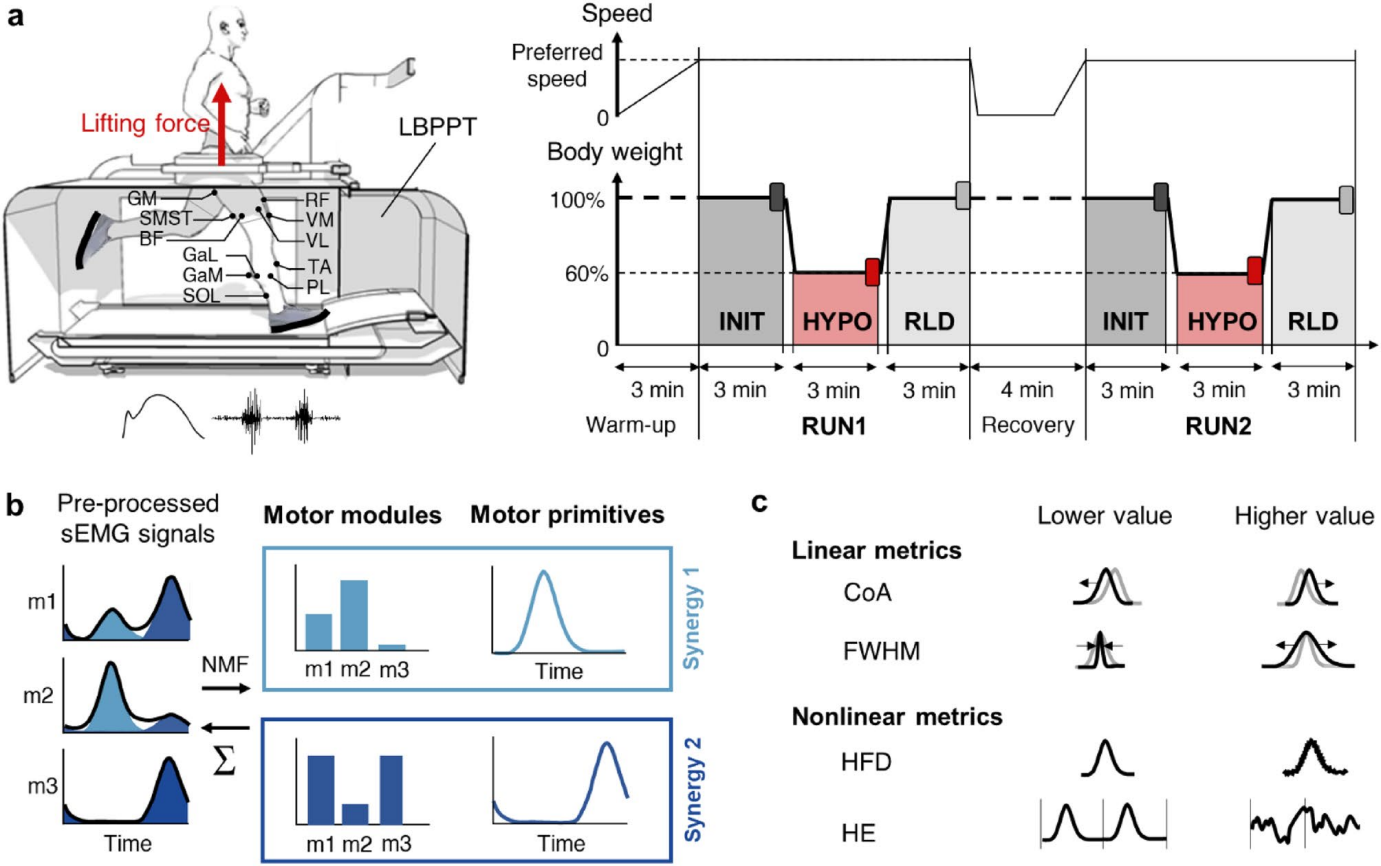
Experimental design and muscle synergy analyses. (**a**) Normal ground reaction force and sEMG activity (11 muscles; GM, gluteus maximus; VM, vastus medialis; VL, vastus lateralis; RF, rectus femoris; STSM, semitendinosus/ semimembranosus; BF, biceps femoris; GaM, gastrocnemius medialis; GaL, gastrocnemius lateralis; SOL, soleus; TA, tibialis anterior; PL, peroneus longus) were collected from healthy men (n = 38) during 2 runs (RUN1 and RUN2) at preferred speed on a LBPPT. Each run consisted of 3 conditions (INIT, HYPO, RLD). The darker rectangles indicate the 60 running cycles considered at the end of each condition. (**b**) Muscle synergies were extracted from pre-processed sEMG signals using NMF. They consisted of time-invariant motor modules activated by time-varying motor primitives, whose linear combination approximated the pre-processed sEMG signals. (**c**) Motor primitives were compared across conditions and runs using linear and nonlinear metrics. The center of activity (CoA) indicated when their main activation occurred in time and the full width at half maximum (FWHM) indicated their duration. The HFD described their local complexity (i.e. roughness within each running cycle) and the HE described their global complexity (i.e. irregularity across running cycles). The explanatory diagrams for the nonlinear metrics are adapted from the work of Santuz et al.^34^.

In preparation for and following the Apollo 11–17 missions (1961–1972), several devices were developed to simulate hypogravity running^4,5^. Among these, the Lower Body Positive Pressure Treadmill (LBPPT) applies a lifting force at the runner’s center of mass^6^ (Fig. 1a). This results in the adoption of a bouncing running pattern along with complex, although understudied, neuromuscular adjustments^7^. In contrast to the expected overall reduction in lower limb muscle surface electromyographic (sEMG) activity^8^, the few available studies reported reduced sEMG activity of most lower limb muscles during the braking phase of the running cycle but of only some of them during the push-off phase^9–11^. They also found unchanged or even increased hamstring sEMG activity during both phases^11–14^. However, they were limited to quantifying the sEMG activity of the lower limb muscles independently of each other.

Electrical microstimulation experiments suggest that the central nervous system may activate groups of functionally related muscles, called muscle synergies, rather than individual muscles^15–18^. This may simplify locomotor control by reducing the number of degrees of freedom of the musculoskeletal system that must be controlled simultaneously^19–21^. Such muscle synergies are usually extracted from sEMG signals using non-negative matrix factorization^22,23^ (NMF), which identifies time-invariant muscle contributions (motor modules) scaled by time-varying coefficients (motor primitives)^24^ (Fig. 1b). Although it depends on the chosen reconstruction quality criterion, four muscle synergies are generally sufficient to account for lower limb muscle sEMG signals during unperturbed running. Each describes a specific phase of the running cycle, namely braking, push-off, early flight, and late flight^24,25^.

To date, no research has examined the muscle synergies inherent in simulated hypogravity running, which substantially reduces the mechanical constraints on the musculoskeletal system. Instead, previous studies have focused on external perturbations such as running on uneven terrain^26,27^, unpredictable terrain^27^, or at extremely high speeds^28^, that make the task more mechanically demanding. They highlighted the consistency of the muscle synergies describing the four phases of the running cycle, with almost unchanged motor modules but adjusted motor primitives. The latter would be systematically wider, i.e. of longer duration relative to the running cycle. This would increase the overlap between temporally adjacent muscle synergies, facilitating the transition from one synergy to another^26–30^.

More advanced nonlinear metrics derived from fractal theory provided a complementary approach to the analysis of motor primitives, considered as self-affine time series^28,31^. Among them, some studies have used the Higuchi’s fractal dimension (HFD) to assess their local complexity, i.e. their roughness within a running cycle^27,28^. Others have used the Hurst exponent (HE) to quantify their global complexity, i.e. is their irregularity across running cycles^29^ (Fig. 1c). Both the local and global complexity of the motor primitives would decrease when running in the presence of the external perturbations mentioned above^27–29^. This suggests that such conditions require a less flexible, i.e. more constrained, locomotor control^32,33^ to maintain functionality.

Here, we sought to better understand how locomotor control adjusts to simulated hypogravity running by examining the muscle synergies in the light of fractal theory. We recorded from 38 heathy men sEMG activity of 11 right lower limb muscles during 3 consecutive running conditions performed at preferred speed on a LBPPT. The initial condition (INIT) was run at 100% body weight (1 g), followed by the simulated hypogravity condition (HYPO) at 60% body weight (0.6 g), and the reloaded condition (RLD) at 100% body weight (1 g). This was repeated twice (RUN1 and RUN2) to account for any familiarization effect. We then extracted muscles synergies from pre-processed sEMG signals using NMF and compared them between conditions and runs using both linear and non-linear metrics (Fig. 1).

We expected a more flexible locomotor control, characterized by increased complexity and reduced width of motor primitives. Instead, we found a specific reorganization of locomotor control in the face of the paradoxical nature of simulated hypogravity running. The reduced mechanical constraints on the musculoskeletal system led to an increased local and global complexity of the motor primitives. However, the increased temporal (shortened stance phase) and sensory (deteriorated proprioceptive feedback) constraints led to a widening of the motor primitives and to an increased hamstring contribution during the stance phase. We concluded that simulated hypogravity running allows for a more flexible locomotor control, which is not free of all constraints.

## Results

### Reported results

Muscle synergies did not differ between RUN1 and RUN2, highlighting the repeatability of locomotor control. Therefore, both runs were combined for subsequent analysis. Muscle synergies also did not differ between INIT and RLD because opposite adjustments occurred from INIT to HYPO and from HYPO to RLD. As a result, only the adjustments from INIT to HYPO will be reported. Those from HYPO to RLD and from INIT to RLD remain visible in the figures, with details in the Supplementary Tables.

### Slowing of stride frequency

**Figure 2:**
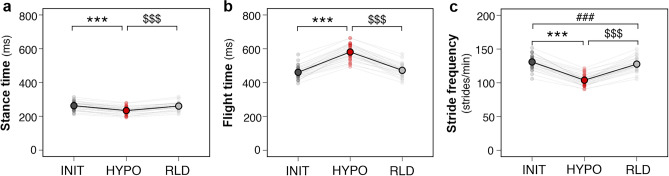
Spatiotemporal parameters of running. A main effect of condition was found for **a** stance time (ANOVA, F_(2, 180)_ = 461, p < 0.001), **b** flight time (ANOVA, F_(2, 181)_ = 280,* p *< 0.001), and **c** stride frequency (ANOVA, F_(2, 180)_ = 913,* p* < 0.001). Data are averaged over all participants (n = 38), with individual data displayed. Significant adjustments determined by post-hoc analysis (multiple comparisons with Tukey’s adjustment) are shown for HYPO *vs*. INIT (*), RLD *vs.* HYPO ($), and RLD vs. INIT (#) (see Supplementary Table 1 for details). The number of symbols indicates the statistical level (three for* p *< 0.001).

The spatiotemporal parameters of running were compared across conditions (Fig. 2). From INIT to HYPO, stance time decreased slightly (−12 ± 5%, effect size (ES) = 1.3) while flight time increased largely (+28 ± 4%, ES = 2.9), resulting in a slowing of stride frequency (−11 ± 3%, ES = 2.4).

### Sharing of similar muscle synergies

**Figure 3:**
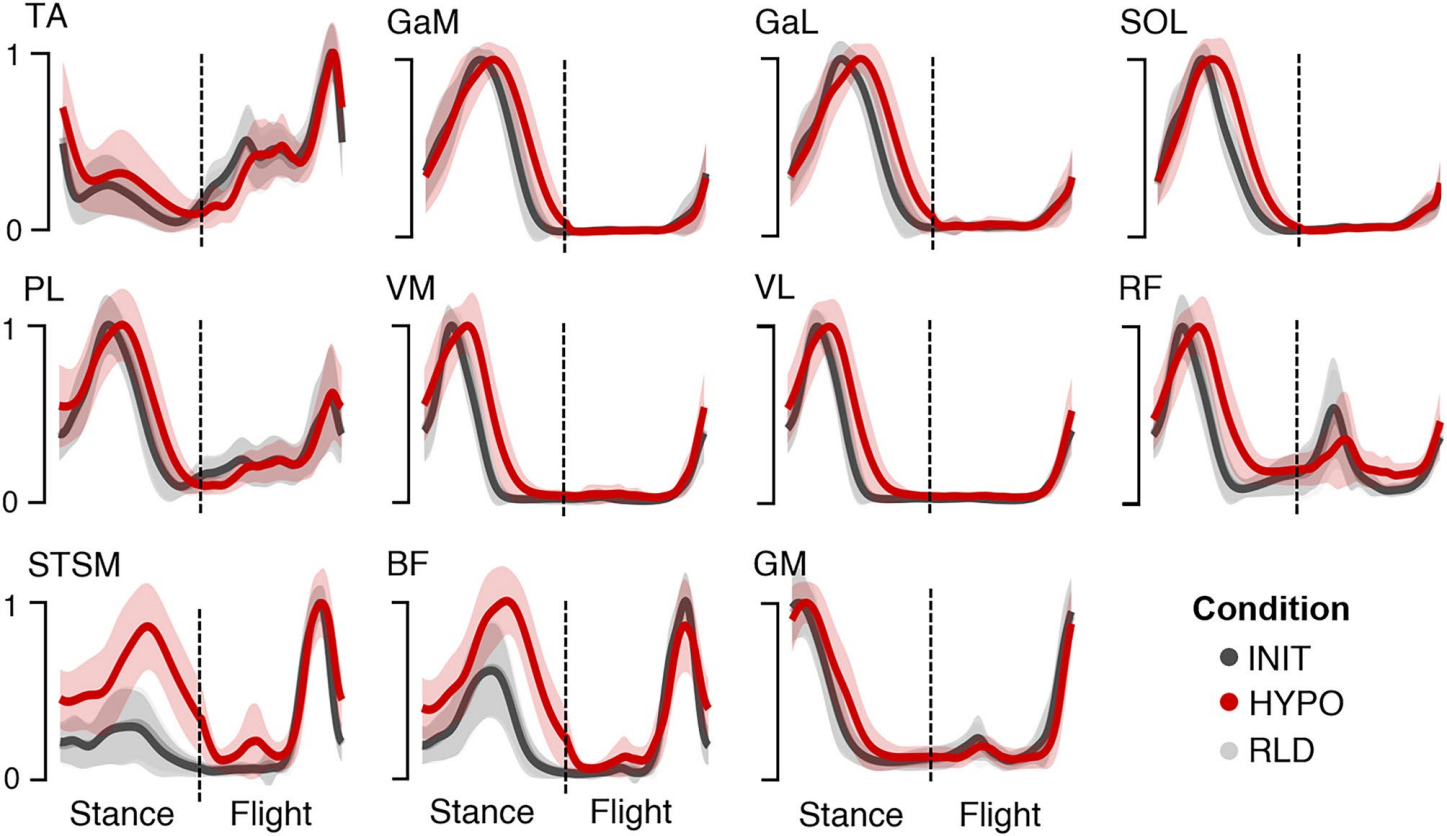
Pre-processed sEMG signals. The x-axis distinguishes the stance phase from the flight phase, normalized to the same number of points. The y-axis shows the normalized sEMG activity. Data are averaged over all participants (n = 38), with shaded standard deviation.

The sEMG signals were pre-processed for the 11 recorded muscles: Gluteus maximus (GM), vastus medialis (VM), vastus lateralis (VL), rectus femoris (RF), biceps femoris (BF), semitendinosus/semimembranosus (STSM), gastrocnemius medialis (GaM), gastrocnemius lateralis (GaL), soleus (SOL), peroneus longus (PL), and tibialis anterior (TA) (Fig. 3). Then, the muscle synergies were extracted using NMF. The minimum number of synergies required to satisfactorily reconstruct the pre-processed sEMG signals (see Methods for threshold criterion) was slightly lower in HYPO than in INIT (3.8 ± 0.1 *vs*. 3.9 ± 0.1, multiple comparisons with Tukey’s adjustment, *p* < 0.05, ES = 0.4). Reconstruction quality, assessed by the coefficient of determination between the original and reconstructed sEMG signals, was high in all conditions (INIT: 0.870 ± 0.003, HYPO: 0.853 ± 0.003, RLD: 0.873 ± 0.003), although moderately lower in HYPO than in INIT (multiple comparisons with Tukey’s adjustment, *p* < 0.001, ES = 0.6) (see Supplementary Table 2 for details on RLD vs. HYPO and RLD vs. INIT).

**Figure 4:**
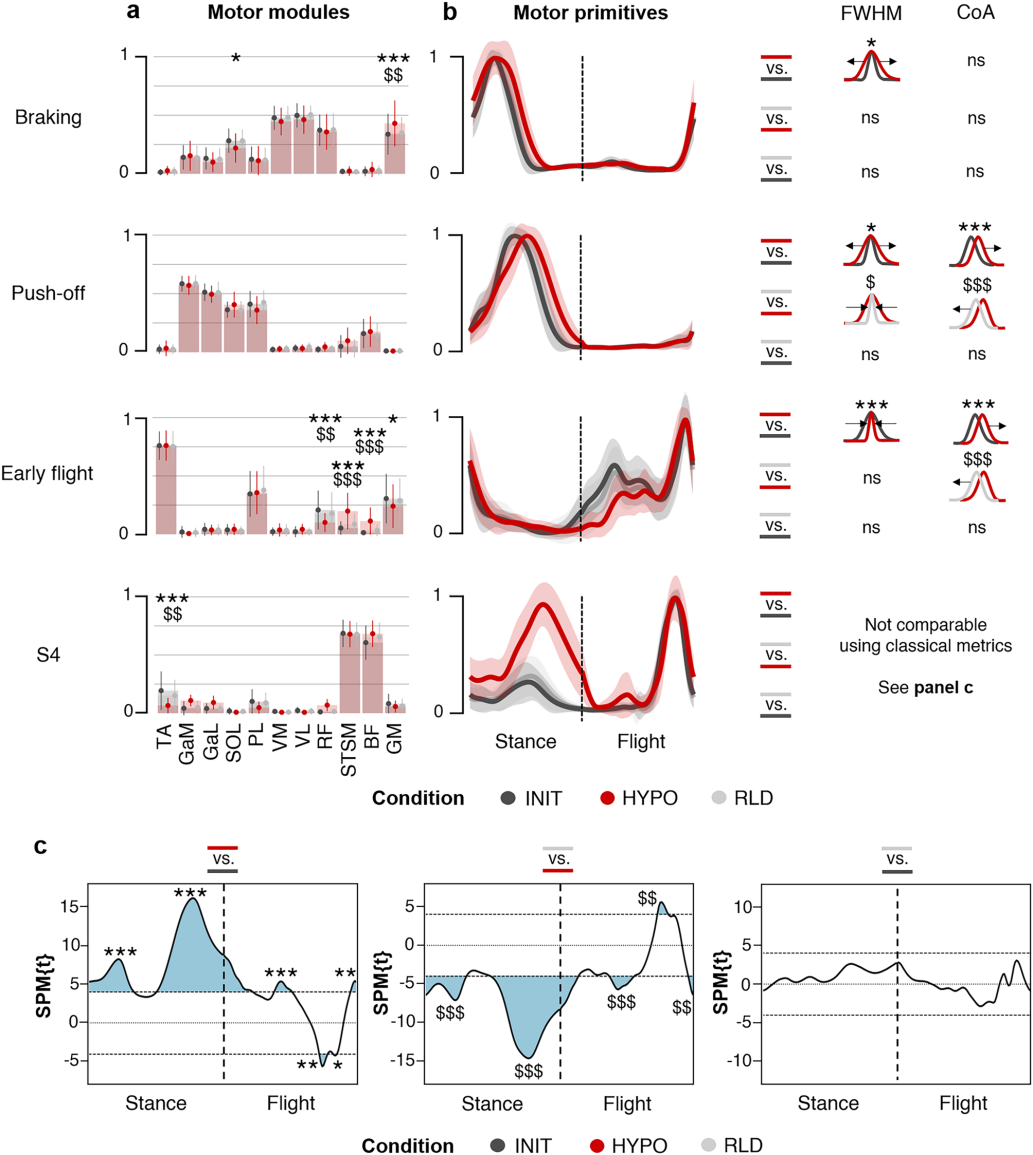
Comparison of muscle synergies across conditions. (**a**) Motor modules. The y-axis shows the normalized muscle contributions, with individual data displayed. A (Condition x Synergy x Muscle) interaction was found (permutation ANOVA, F_(60, 8491)_ = 4.6,* p* < 0.001). (**b**) Motor primitives. The x-axis distinguishes the stance phase from the flight phase, normalized to the same number of points. The y-axis shows the normalized amplitude with shaded standard deviation. (Condition x Synergy) interactions were found for the FWHM (permutation ANOVA, F_(4, 576)_ = 7.6,* p* < 0.001) and the CoA (ANOVA, F_(4, 576)_ = 5.1,* p* < 0.001). **c** S4 could not be compared using classical metrics (COA and FWHM), but SPM revealed a main effect of the condition (SPM ANOVA, F* = 8.3). The blue shaded supra-threshold clusters (t* = 4.02) correspond to the time periods in which the S4 motor primitives differed. Motor modules and primitives are averaged across participants presenting the synergy of interest (see Supplementary Table 3). Significant adjustments determined by *post-hoc* analysis (multiple comparisons with Holm’s adjustment for panels a-b and with Bonferroni’s adjustment for panel c) are shown for HYPO *vs*. INIT (*) and RLD *vs.* HYPO ($) (see Supplementary Table 4–5 for details). The number of symbols indicates the statistical level (one for* p* < 0.05, two for* p* < 0.01, three for* p* < 0.001).

K-means clustering identified four muscle synergies across all three conditions (Fig. 4). The first synergy was functionally related to the braking phase: Its main activation occurred during the first half of the stance phase, with a major contribution from the hip and knee extensors muscles (GM, VM, VL and RF). The second synergy described the push-off phase: Its main activation occurred during the second half of the stance phase, with a major contribution from the ankle plantarflexor muscles (GaM, GaL, SOL and PL). The third synergy was related to the early flight phase, with a major contribution from the ankle dorsiflexor (TA) and evertor (PL) muscles. The fourth synergy differed greatly between conditions: In INIT and RLD, it was functionally related to the late flight phase (preactivation before impact), whereas in HYPO it presented an additional activation during the push-off phase. In all conditions, it showed a major contribution from the hamstring muscles (BF and STSM). In the following, we will refer to this fourth synergy as “S4”. Note that some participants did not show all four muscle synergies mentioned above (Supplementary Table 3).

### Increased hamstring contribution

The motor modules were first compared between INIT and HYPO by examining the muscle contributions (Fig. 4a). During the braking phase, the SOL contribution decreased (−22 ± 27%, ES = 0.5), but the GM contribution increased (+29 ± 18%, ES = 0.5). No difference was found in the muscle contributions during the push-off phase. However, the S4 motor primitives showed an additional activation during this phase (see Fig. 4c for detailed analysis), highlighting a new contribution of the hamstring muscles (STSM and BF) to this phase. During the early flight phase, the RF and GM contributions decreased (RF: −51 ± 17%, ES = 0.7 and GM: −23 ± 27%, ES = 0.3), but the hamstring contribution increased (STSM: +268 ± 12%, ES = 1.1 and BF: +795 ± 18%, ES = 1.0). The S4 motor modules showed a decreased TA contribution (−67 ± 16%, ES = 0.9).

**Figure 5:**
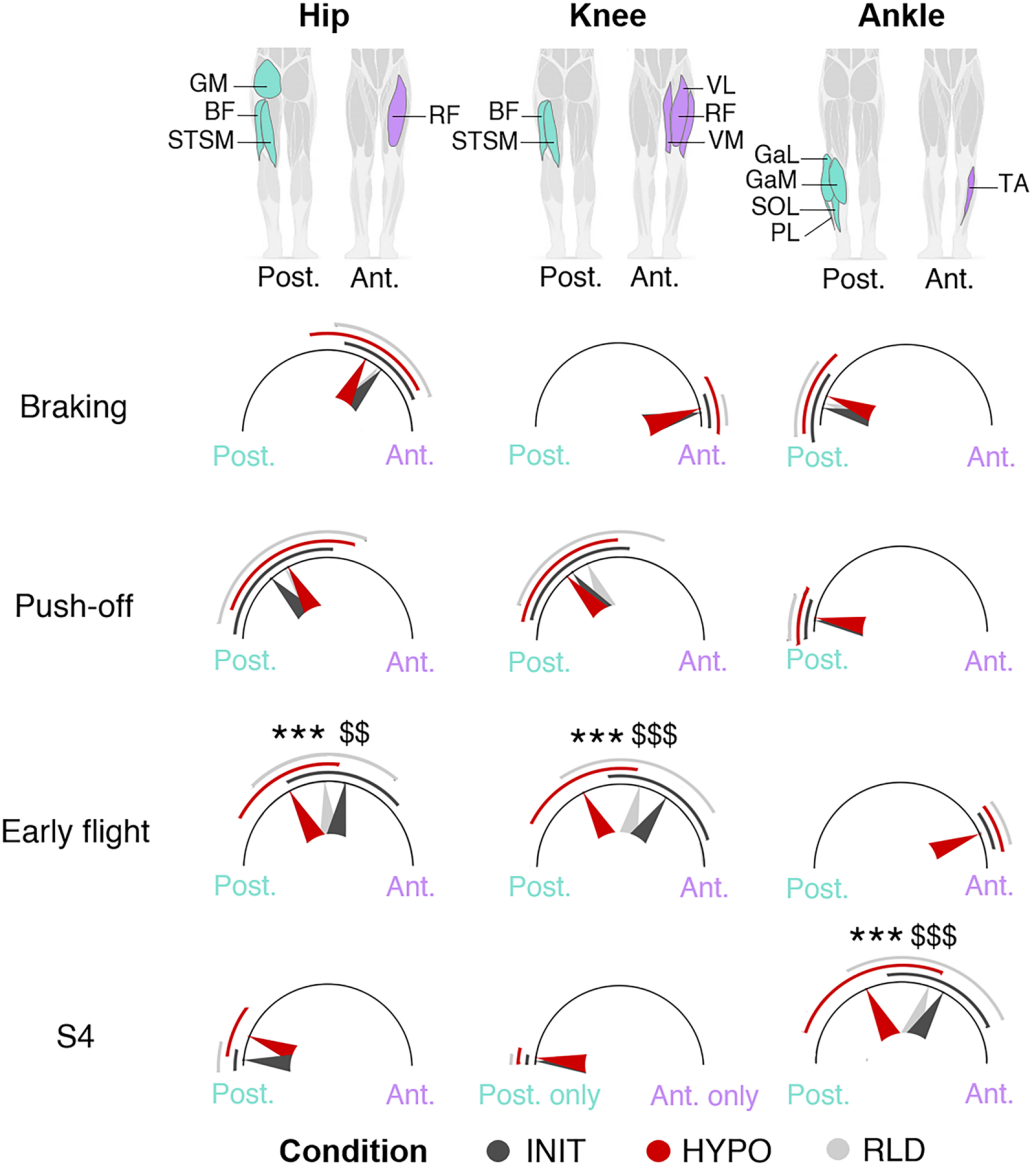
Co-contribution from anterior and posterior lower limb muscles. For each joint, the anterior (purple) and posterior (green) muscles considered are shown in the header. Arrows pointing to the right indicate a greater contribution from anterior muscles while arrows pointing to the left indicate a greater contribution from posterior muscles. Arrows pointing upwards indicate a perfect balance between the contribution of the anterior and posterior muscles. An interaction (Condition x Synergy x Joint) was found (permutation ANOVA, F(12, 2313) = 11,* p* < 0.001). The CI is averaged across participants presenting the synergy of interest (see Supplementary Table 3). The standard deviation is shown with curved lines. The significant adjustments determined by *post-hoc* analysis (multiple comparisons with Holm’s adjustment) are shown for HYPO *vs*. INIT (*) and RLD *vs.* HYPO ($) (see Supplementary Table 5 for details). The number of symbols indicates the statistical level (three for* p* < 0.001).

They were then compared by calculating a co-contribution index (CI), which assessed the proportion of contributions from the anterior and posterior lower limb muscles at the three joints considered (hip, knee and ankle) (Fig. 5, see Methods for details). From INIT to HYPO, the CI decreased at the hip and knee joints during the early flight phase (−37 ± 6%, ES = 1.0 and −48 ± 5%, ES = 1.3, respectively), indicating an increased contribution of the hamstrings at the expense of the quadriceps. It also decreased at the ankle joint in the S4 motor modules (−46 ± 6%, ES = 1.1), indicating an increased contribution of the triceps surae at the expense of the TA.

### Widening of stance motor primitives

Motor primitives were compared across conditions using linear metrics. Specifically, their center of activity (CoA) and full width at half maximum (FWHM) were calculated. They provided an indication of when their main activation occurred in the running cycle and their duration, respectively (Fig. 4b). From INIT to HYPO, the push-off and early flight motor primitives were delayed in the running cycle (higher CoA; +20 ± 23%, ES = 1.3 and +9 ± 14%, ES = 0.7, respectively). Furthermore, the braking and push-off motor primitives were of longer duration relative to the running cycle (higher FWHM; +17 ± 35%, ES = 0.8 and +13 ± 35%, ES = 0.8, respectively), while the early flight primitive was of shorter duration (lower FWHM; −20 ± 26%, ES = 0.4).

The S4 motor primitives differed greatly between INIT and HYPO, with their additional activation in the push-off phase giving them a bimodal profile in HYPO. As they could not be compared using classical metrics, they were excluded from the CoA and FWHM analysis. Instead, the S4 motor primitives were compared across conditions using one-dimensional statistical parametric mapping^35^ (SPM) (Fig. 4c). This allowed the calculation of the t-statistics, denoted SPM{t}, for each time point of the running cycle (from 1 to 200). When SPM{t} exceeded the critical threshold computed using random field theory (t*), the motor primitives were considered to be significantly different. From INIT to HYPO, the SPM analysis mainly highlighted the aforementioned additional activation of the S4 motor primitives during the push-off phase (between time points 52–122).

**Figure 6:**
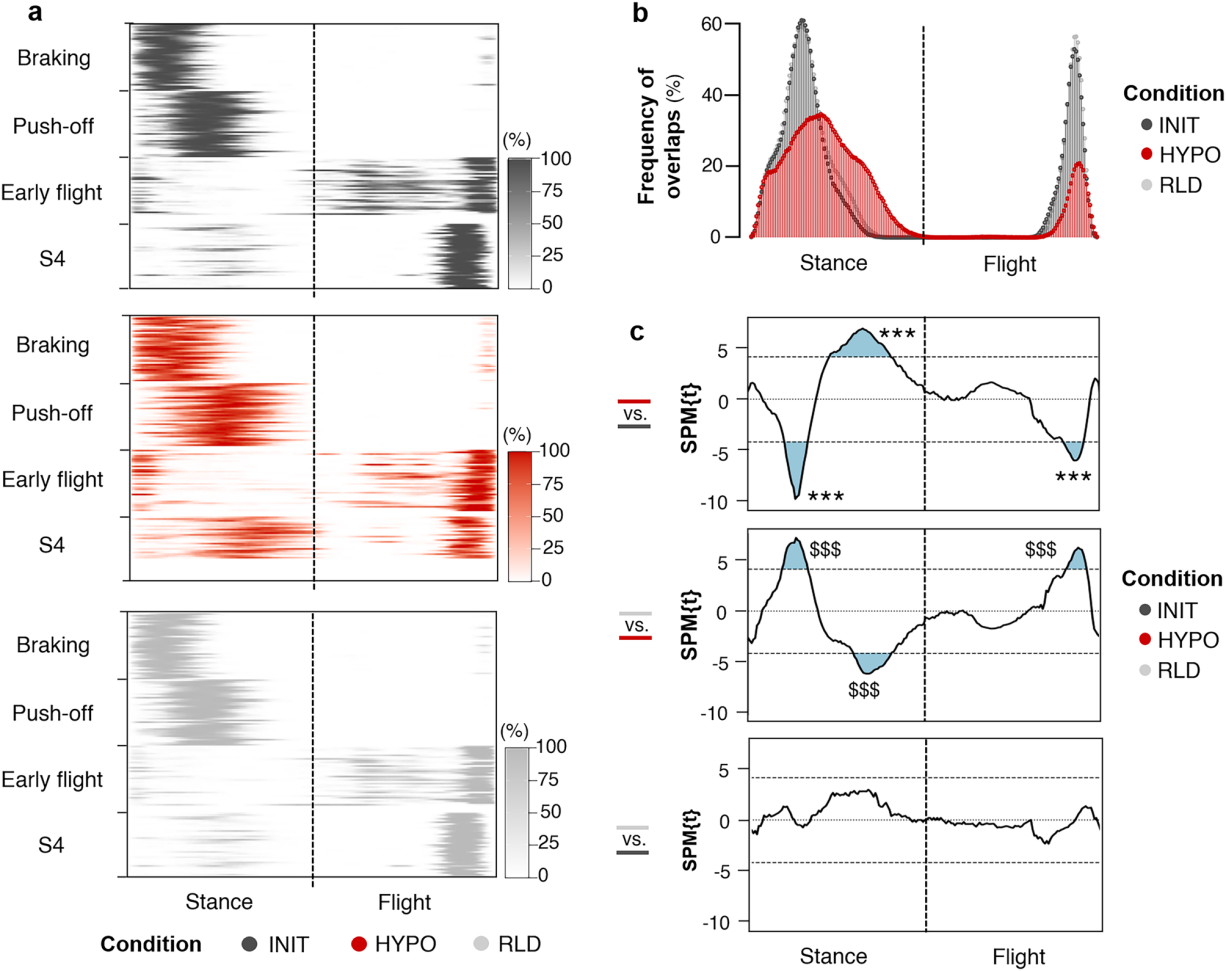
Overlaps of motor primitives. (**a**) Frequency at which the motor primitives exceeded half of their maximum. Each row of the heatmaps corresponds to a participant, each column to a time point. The results are color-coded: from white (the primitives never exceeded half of their maximum) to the darkest color (the primitives exceeded half of their maximum in all running cycles considered). Missing motor primitives are reported as fully white rows (participants did not systematically present the four muscle synergies, see Supplementary Table 3). (**b**) Frequency of overlaps between motor primitives. The x-axis distinguishes the stance phase from the flight phase, normalized to the same number of points. (**c**) The frequency of overlaps was compared using SPM, which detected a main effect of the condition (SPM ANOVA, F* = 9.4). The blue shaded supra-threshold clusters (t* = 4.2) correspond to the time periods in which the frequency of overlaps differed. The significant adjustments determined by *post-hoc* analysis (multiple comparisons with Bonferroni’s adjustment) are shown for HYPO *vs*. INIT (*) and RLD *vs.* HYPO ($). The number of symbols indicates the statistical level (three for*p* < 0.001).

The frequency at which the motor primitives exceeded half of their maximum of the 60 running cycles considered was computed for each time point of the running cycle (Fig. 6a). This enabled to calculate the frequency of overlaps (Fig. 6b), which we compared between conditions using SPM (Fig. 6c): An overlap occurs when at least two primitives simultaneously exceed half of their maximum. Consistent with the aforementioned widening of the braking and push-off motor primitives, the heat maps showed that both exceeded half of their maximum over a larger range of the running cycle in HYPO than in INIT and RLD. However, these adjustments were not systematic over the 60 running cycles considered, as shown by the lighter shades of grey and red on the heat maps. This resulted in a decreased frequency of overlaps between the braking and push-off motor primitives (between time points 22–34). On the other hand, the heat maps highlighted the additional activation of the S4 motor primitives during the push-off phase in HYPO, leading to an increased frequency of overlaps between the push-off and S4 motor primitives (between time points 48–81).

### Increased local and global complexity of motor primitives

**Figure 7:**
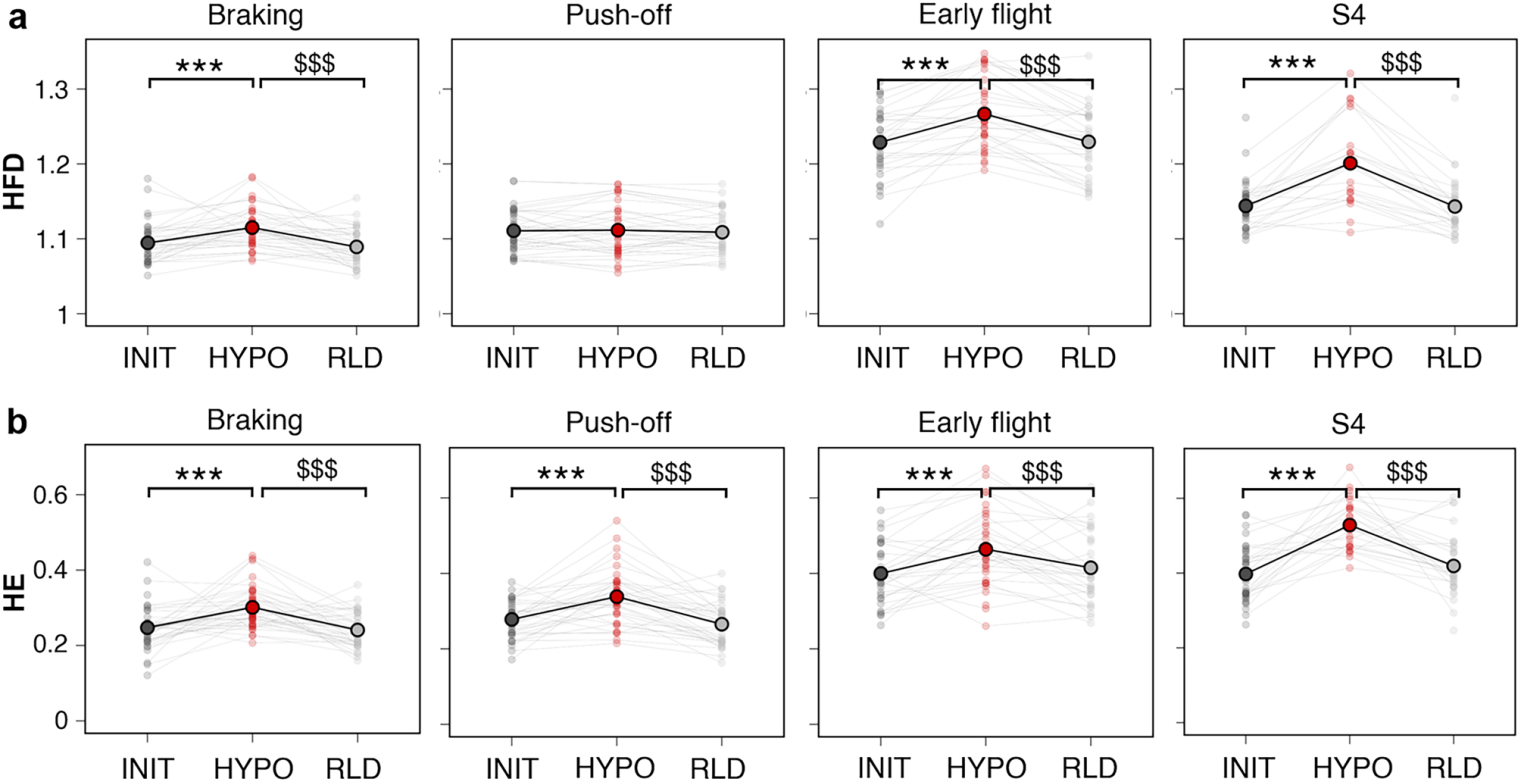
Local and global complexity of motor primitives. (Condition x Synergy) interactions were found for (**a**) the HFD (ANOVA, F(6,747) = 14, p < 0.001) and (**b**) the HE (ANOVA, F(6,7450) = 3.4, p < 0.01) of motor primitives. Data are averaged across participants presenting the synergy of interest (see Supplementary Table 3), with individual data displayed. Significant adjustments determined by *post-hoc* analysis (multiple comparisons with Holm’s adjustment) are shown for HYPO *vs*. INIT (*), RLD *vs.* HYPO ($) (see Supplementary Table 5 for details). The number of symbols indicates the statistical level (three for *p *< 0.001).

Finally, the motor primitives were compared across conditions using nonlinear metrics derived from fractal theory. Specifically, their Higuchi’s fractal dimension (HFD) and their Hurst exponent (HE) were computed. They describe their roughness within each running cycle and their irregularity across running cycles, respectively (Fig. 7). From INIT to HYPO, the HFD increased moderately for the braking and early flight motor primitives (+2 ± 24% and +3 ± 15%, ES = 0.7 for both), and strongly for the S4 motor primitives (+5 ± 9%, ES = 1.5). It did not vary for the push-off primitives (Fig. 7a). On the other hand, the HE increased for all motor primitives, but moderately for the braking and push-off primitives (+23 ± 22%, ES = 0.9 and +21 ± 23%, ES = 0.8, respectively), slightly for the flight primitives (+14 ± 25%, ES = 0.5), and strongly for the S4 primitives (+34 ± 12%, ES = 1.7) (Fig. 7b).

### Correlations between adjustments in the spatiotemporal parameters of running and motor primitives

**Figure 8:**
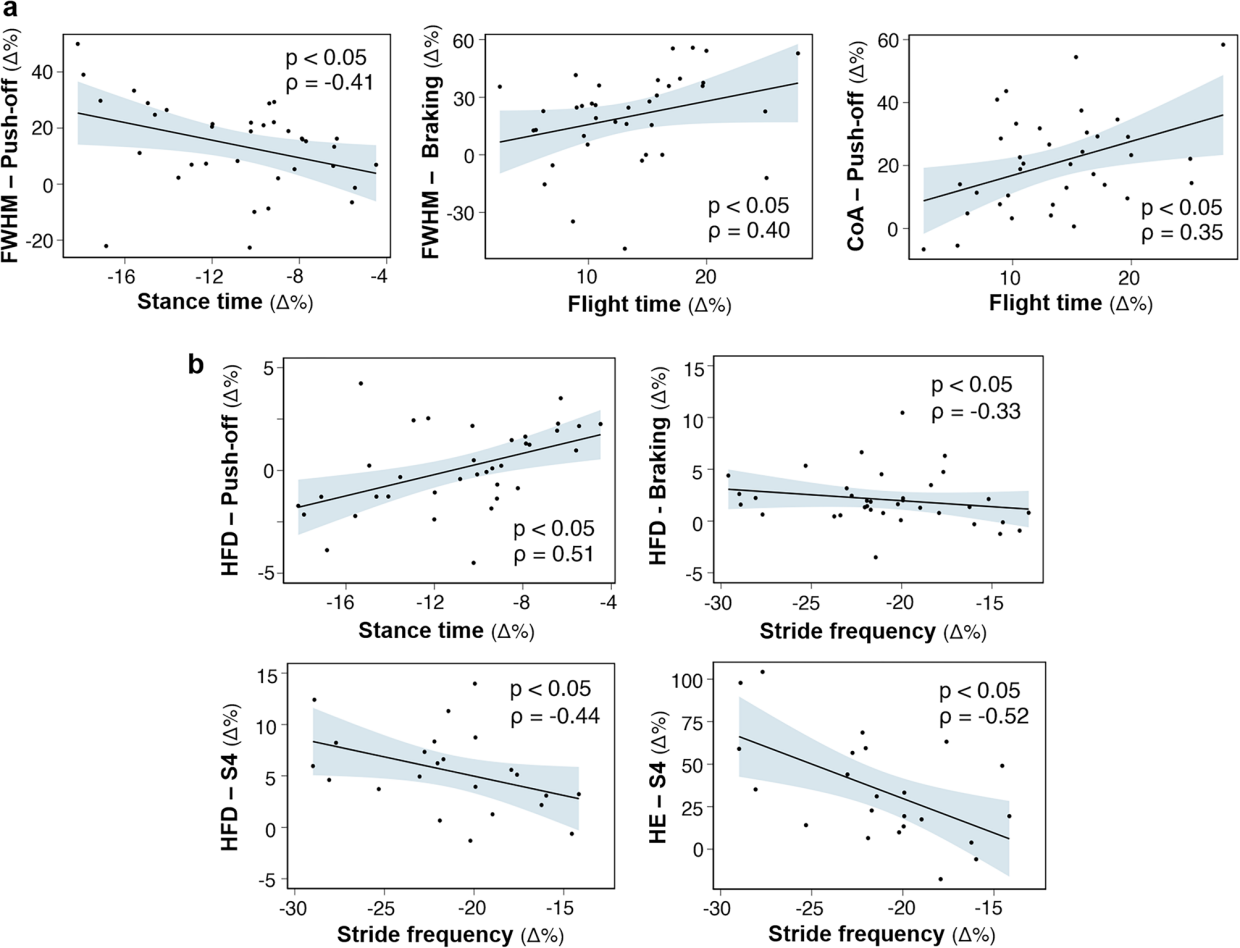
Correlation analysis of relative adjustments from INIT to HYPO. (**a**) Correlations between relative adjustments in spatiotemporal parameters of running and in motor primitives, as measured by linear metrics. (**b**) Correlations between relative adjustments in spatiotemporal parameters of running and in motor primitives, as measured by nonlinear metrics. Spearman’s rank correlations were performed on the data from the participants presenting the synergy of interest (see Supplementary Table 3). The blue shaded areas correspond to the 95% confidence interval. Non-significant correlations are shown in Supplementary Table 6.

Finally, the adjustments in the spatiotemporal parameters of running (stance time, flight time, stride frequency) were correlated with those in motor primitives (FWHM, COA, HFD, HE) (Fig. 8). When focusing on linear metrics, correlation analysis showed that the participants with the greatest decrease in stance time had the greatest increase in the FWHM of the push-off primitives (ρ = −0.41, *p* < 0.05). Furthermore, those with the greatest increase in flight time had the greatest increase in the FWHM of the braking primitives and in the CoA of the push-off primitives (ρ = 0.40 and ρ = 0.35, *p* < 0.05 for both) (Fig. 8a). When looking at the non-linear metrics derived from fractal theory, correlational analysis showed that the participants with the smallest decrease in stance time had the greatest increase in the HFD of the push-off primitives (ρ = 0.51, *p* < 0.05). Finally, those with the greatest decrease in stride frequency had the greatest increase in the HFD of the braking and S4 primitives (ρ = −0.33 and ρ = −0.44, *p* < 0.05 for both), and in the HE of the S4 primitives (ρ = −0.52, *p* < 0.05) (Fig. 8b).

## Discussion

The present study provided a better understanding of locomotor control during simulated hypogravity running. As a preamble, simulated hypogravity running resulted in the adoption of a bouncing running pattern, characterized by a slowing of stride frequency. Although the gravity simulated in this study (0.6 g) is closer to that of Mars than that of the Moon, these biomechanical adjustments are largely consistent with videos from the Apollo 11–17 missions, which showed that the astronauts neither walked nor ran, but rather adopted hopping, skipping^36^ or loping^37^ gaits. This suggests a specific reorganization of locomotor control, which we investigated by analyzing the muscle synergies in the light of fractal theory.

The extracted muscle synergies described the four usual phases of the running cycle, both in normogravity and in simulated hypogravity. In this sense, it has been reported that the postural and walking muscle synergies were consistent up to 0.25 g and 0.16 g, respectively^38,39^. This suggests that the training protocols that astronauts undergo in normogravity (on Earth) may be transferable to hypogravity (e.g., on the Moon or Mars) and vice versa. However, it should be noted that three muscle synergies would be sufficient to account for the sEMG signals when walking in very low gravity (0.07 g), compared to four at higher gravities (from 1 to 0.16 g)^39^. Although the current study only examined the running muscle synergies at 0.6 g, which is far from the gravity of Mars and the Moon, it highlighted some adjustments in the motor modules and motor primitives that should be considered in anticipation for future space missions.

Simulated hypogravity running led to a redistribution of muscle contributions within the motor modules. During the braking phase, the reduced SOL contribution is consistent with its antigravity function^40^. In the S4 motor modules, the decreased TA contribution is attributed to the shift toward a forefoot strike pattern^41,42^. Importantly, the push-off phase, showed no change in the contribution of the ankle plantarflexors (SOL, GaM, GaL, PL), but an increased contribution of the hamstring muscles (BF and STSM), as evidenced by the additional activation of the S4 motor primitive. This was previously reported during simulated hypogravity walking^43^ and confirms our recent findings of increased hamstring activity during simulated hypogravity running, especially in the braking (BF: + 44 ± 18%) and push-off (BF: + 49 ± 12%, STSM: + 123 ± 14%) phases^11^. This may have compensated for the disadvantageous force output of the gastrocnemii in a forefoot versus rearfoot strike pattern^44^. In addition, the hamstring muscle group is known to be involved in increasing walking and running speed^45,46^. Its increased contribution may thus have contributed to accelerating the knee joint flexion of the swing leg, in an attempt to counteract the slowing of stride frequency, characteristic of simulated hypogravity running. This is supported by its increased contribution in the early flight phase, which came at the expense of the RF contribution due to reciprocal inhibition^47^. However, the adjustments in hamstring contribution were not correlated with those in stride frequency. Further studies should therefore investigate the functional role of this muscle group during simulated hypogravity running in order to provide recommendations for pre-flight training of astronauts. As things stand, specific strengthening of this muscle group may be recommended.

Simulated hypogravity running resulted in wider stance motor primitives (higher FWHM of the braking and push-off primitives). Furthermore, the push-off primitives were shifted later in the running cycle (higher CoA). Such adjustments of the stance primitives has previously been observed in genetically modified mice lacking muscle spindles and Golgi tendon organs when compared to wild type^34,48^. These results could therefore be attributed to the deteriorated proprioceptive feedback caused by simulated hypogravity^49,50^. Yet, the opposite adjustments found for early flight motor primitives, which were narrower, rather suggests the influence of temporal constraints: The stance phase was shortened during simulated hypogravity running, with the push-off phase being more affected than the braking phase^11^, while the flight phase was prolonged. This is supported by the correlation analysis, which showed that the participants with the greatest decrease in stance time were those with the greatest widening of the push-off motor primitives. Thus, the lack of time to organize locomotor control during the stance phase may have been compensated for by increasing the overlap between the temporally adjacent braking and push-off motor primitives. Typically reported in the presence of external perturbations^26–30^, such adjustment was not systematically observed across running cycles, as indicated by the decreased frequency of overlaps. This is a first indication that simulated hypogravity running may constrain locomotor control, while allowing its flexibility.

The nonlinear analysis of the motor primitives supported this assumption of a higher locomotor control flexibility. On the one hand, three of the four primitives (those of braking, early flight, and S4) were more locally complex (higher HFD). This result may seem surprising given the literature on externally perturbed locomotion, which reports a decrease in the HFD of motor primitives^27,28^. However, it was expected since simulated hypogravity running is certainly perturbed, but also less mechanically contrained. Only the push-off primitives were not more locally complex. This is again attributed to the aforementioned high temporal constraints on push-off phase^11^. Indeed, the correlation analysis showed that participants with the greatest decrease in stance time were those showing the lowest increase in the HFD of the push-off motor primitives. On the other hand, all motor primitives were more globally complex (higher HE) during simulated hypogravity running. Since the HE increased from 0 (sinusoidal time series) to 0.5 (random time series), it follows that the motor primitives were more irregular across running cycles in simulated hypogravity than in normogravity. This suggests that participants exerted less stride-to-stride control^51^. The unintended horizontal support provided by the LBPPT chamber^52^, in addition to the expected vertical support, could explain this reduced control effort. It would therefore be relevant to study its influence on muscle synergies. In any case, this is in line with the ‘minimum intervention principle’ used by humans to regulate locomotion: Deviations are corrected only when they interfere with task performance^53^.

Is this increase in local and global complexity of motor primitives beneficial for simulated hypogravity running? Correlation analysis showed that participants with the greatest increase in the HFD of the braking and S4 motor primitives, and in the HE of the S4 motor primitives had the greatest decrease in stride frequency. All correlations were moderate to large, indicating a non-negligible relationship between these nonlinear metrics and stride frequency. Because it is systematically modified, stride frequency appears to be the spatiotemporal parameter that best describes the degree of adjustment to simulated hypogravity running. In this sense, it has been proposed that in mature and healthy biological systems, an optimal amount of variability reflects the flexibility of locomotor control to perturbations^32,33^. Less than optimal variability makes the system overly rigid, while greater than optimal variability makes the system overly noisy and unstable. Thus, the higher local and global complexity of the motor primitives observed in this study would reflect a more flexible locomotor control, allowing participants to optimally adjust to simulated hypogravity running. This finding may pave the way for the development of a feedback tool to improve astronauts’ ability to adjust to hypogravity.

Overall, our results revealed a specific reorganization of locomotor control in the face of the paradoxical nature of simulated hypogravity running, which combines reduced mechanical constraints with increased temporal and sensory constraints. With human space exploration back in the spotlight, they deserve to be verified in simulated gravities closer to those of Mars or the Moon. They also need be confirmed in actual hypogravity: Subtle but systematic differences in the running pattern are known, suggesting potential differences in locomotor control^54^. In any case, the current results provide a first insight into locomotor control during simulated hypogravity running. They are a first step towards improving pre-flight training, per-flight countermeasures and post-flight rehabilitation of astronauts.

## Methods

### Participants

Thirty-eight recreational runners (age: 19 ± 1 years old [mean ± sd]; height: 177 ± 6 cm; mass: 69.3 ± 8.1 kg) volunteered for this study. We ensured that they had not suffered any musculoskeletal injury for more than 1 year, and had no history of any neurological impairment. All procedures were approved by the National Ethics Committee for Research in Sports Sciences (reference number: CERSTAPS IRB00012476-2021-31-03-96). All methods were carried out in accordance with the Declaration of Helsinki, and written informed consent was obtained from all participants prior to the experiment.

### Experimental design

Participants were asked to run on a Lower Body Positive Pressure Treadmill (LBPPT) (VIA400X AlterG®, Fremont, CA, USA) at their preferred speed (3.0 ± 0.2 m s^-1^). They were instructed to remain in the center of its chamber to minimize involuntary horizontal support^52^. The preferred speed was self-selected while running at 100% body weight (1 g) during a familiarization session performed the week pior to the experimental protocol. After a 3-min warm-up to gradually reach the preferred speed, the experimental protocol consisted of two 9-min runs separated by a 4-min recovery period. Each run included 3 consecutive 3-min running conditions: The initial (INIT) and reloaded (RLD) conditions were run at 100% body weight, while the intermediate simulated hypogravity condition (HYPO) was run at 60% body weight (0.6 g). The transitions phases between the conditions were progressive and lasted 12 ± 2 s.

### Data recordings

The normal component of the ground reaction force was recorded using instrumented insoles (Loadsol, Novel®, Munich, Germany; 100 Hz) placed in standardized running shoes (Run active, Kalenji®). Surface electromyographic (sEMG) activity was recorded from 11 muscles of the right lower limb using bipolar electrodes (miniWave COMETA®, Milan, Italy; 2000 Hz), including tibialis anterior (TA), gastrocnemius medialis (GaM), gastrocnemius lateralis (GaL), soleus (SOL), peroneus longus (PL), vastus medialis (VM), vastus lateralis (VL), rectus femoris (RF), biceps femoris (BF), semitendinosus and semimembranosus (STSM), and gluteus maximus (GM). Skin preparation and electrode placement followed the recommendations of the Surface Electromyography for the Non-Invasive Assessment of Muscles (SENIAM) project^55^. To reduce motion artefacts, the electrodes were wrapped with a self-adhesive and a tubular bandage around the thigh and shank. The recorded force and sEMG signals were synchronized. All data analyses were performed on the last 60 running cycles of each condition.

### Spatiotemporal parameters of running

Touchdown and toe-off were identified from the normal component of the ground reaction force using a threshold of 50 N. Stance time, flight time and stride frequency were calculated. The foot strike pattern (rearfoot or midfoot/forefoot) was obtained from sensors located in the anterior and posterior regions of the instrumented insoles.

### EMG pre-processing

The raw sEMG signals were first band-pass filtered (cutoff at 10 and 500 Hz). They were then high-pass filtered (cutoff at 50 Hz), full-wave rectified, and low-pass filtered (cutoff at 20 Hz) using a 4th order IIR Butterworth zero-phase filter^24^. After subtracting the minimum, the sEMG signals were normalized to the maximum activity recorded for every muscle in each running condition^56^. Each running cycle was then time-normalized to 200 points, assigning 100 points to the stance phase and 100 points to the flight phase.

### Muscle synergy extraction

Muscle synergies were extracted from the pre-processed sEMG signals using a custom R script (v3.6.3, R Foundation for Statistical Computing, Vienna, Austria). The m = 11 muscles listed above were included in the analysis (TA, GaM, GaL, SOL, PL, VM, VL, RF, BF, STSM, GM). For each condition and participant, the m = 11 time-dependent muscle activity vectors were grouped into a matrix V with dimensions m = 11 rows and n = 1200 columns (200 points × 60 running cycles). The matrix V was factorized using the classical Gaussian Non-negative Matrix Factorization (NMF) algorithm^22,57^ such that V ≈ V_*R*_ = WH. The new matrix V_*R*_, reconstructed by multiplying the matrices W and H, approximates the initial matrix V. The motor modules matrix W contained the time-invariant muscle weights that describe the relative contribution of each muscle within a given synergy. It had dimensions *m* × r, where “r” is the minimum number of synergies necessary to satisfactorily reconstruct the original matrix V. The motor primitive matrix H, with dimensions *s* × *n*, contained the time-dependent activation of each synergy. The update rules for W and H are presented in Eqs. (1) and (2).

1 $${\text{H}}_{{{\text{i}} + 1}} = {\text{H}}_{{\text{i}}} \odot \frac{{{\text{W}}_{{\text{i}}}^{{\text{T}}} {\text{V}}}}{{{\text{W}}_{{\text{i}}}^{{\text{T}}} {\text{W}}_{{\text{i}}} {\text{H}}_{{\text{i}}} }}$$

2 $${\text{W}}_{{{\text{i}} + 1}} = {\text{W}}_{{\text{i}}} \odot \frac{{{\text{V }}\left( {{\text{H}}_{{{\text{i}} + 1}} } \right)^{{\text{T}}} }}{{{\text{W}}_{{\text{i}}} {\text{H}}_{{{\text{i}} + 1}} \left( {{\text{H}}_{{{\text{i}} + 1}} } \right)^{{\text{T}}} }}$$

where “T” is the transposed matrix and $\odot$ the element-by-element multiplication.

The quality of the reconstruction was assessed by calculating the coefficient of determination (R^2^) between the original and reconstructed matrices (V and V_R_, respectively), as shown in Eqs. (3) and (4).

3 $${{\text{R}}}^{2}=1- \frac{{\text{SSE}}}{{\text{SST}}}$$

4 $${\text{SSE}}= \sum_{\begin{array}{c}1 \le i \le m\\ 1 \le j \le n\end{array}}{\left({{\text{V}}}_{{\text{i}},{\text{j}}} -{{{\text{V}}}_{{\text{R}}}}_{{\text{i}},{\text{j}}}\right)}^{2} ;\mathrm{ SST}= \sum_{\begin{array}{c}1 \le i \le m\\ 1 \le j \le n\end{array}}{\left({{\text{V}}}_{{\text{i}},{\text{j}}} - \overline{{\text{V}} }\right)}^{2}$$

where SSE is the sum squared error, SST the sum squared total and $\overline{{\text{V}} }$ the average of the matrix V.

Iterations were stopped when the change in the calculated R^2^ was less than 0.01% over the last 20 iterations^24^. This operation was first completed by setting the number of synergies to 1. It was then repeated by increasing the number of synergies, up to a maximum of 8 synergies. The number of 8 was chosen to be equal to 75% of the number of monitored muscles, as extracting a number of synergies equal to the number of monitored muscles would not reduce the dimensionality of the data. For each number of synergies, the factorization was repeated 5 times, each time generating new randomized initial matrices W and H, to avoid local minima^58^. The solution with the highest R^2^ was then selected. To select the minimum number of synergies required to reconstruct the original sEMG signals, the curve of R^2^ values *vs.* number of synergies was fitted using a simple linear regression model. The mean squared error between the curve and the linear regression was calculated^59^. The lowest abscissa point was then removed and the error between this new curve and its new linear regression was calculated. This was repeated until there were 2 points left on the curve or the mean squared error was less than 10^–4^. This was done to find the most linear part of the curve of R^2^ values *vs.* number of synergies, assuming that in this section the reconstruction quality could not be significantly increased by adding more synergies to the model.

### Linear metrics for the analysis of muscle synergies

#### Co-contribution index

For each lower limb joint (hip, knee, ankle), we calculated a co-contribution index (CI) from the motor modules. To do this, we computed the mean contributions of the anterior ($\overline{{\text{ant}} }$) and posterior ($\overline{{\text{post}} }$) mobilizing muscles^60^. For the hip, the anterior mobilizing muscle was the RF anterior and the posterior was the GM. For the knee, the anterior mobilizing muscles were RF, VL and VM and the posterior were the STSM and BF. For the ankle, the anterior mobilizing muscle was the TA and the posteriors were the GaM, GaL, SOL and PL. Equation (5) defines the CI.

5 $${\text{CI}}= \frac{\overline{{\text{ant}}} }{\overline{{\text{ant}} }+ \overline{{\text{post}}} }$$

According to this definition, (i) ${\text{CI}}$= 0 when only the posterior muscles contribute to the considered joint; (ii) ${\text{CI}}$= 1 when only the anterior muscles contribute and (iii) CI = 0.5 if anterior and posterior muscles contribute equally.

#### Center of activity of motor primitives

We compared the averaged motor primitives (over the 60 considered running cycles) by evaluating first their center of activity (CoA). The CoA is an indication of when their main activation happens in time. It was calculated as the angle of the vector (in polar coordinates) oriented towards the center of mass of that circular distribution^61^. The polar direction represented the running cycle’s phase, 0° corresponding to the touchdown and 360° to the next touchdown. Equations (6), (7) and (8) define the CoA.

6 $$A = \mathop \sum \limits_{t = 1}^{p} \left( {\cos \theta_{t} \times \overline{H}_{t} } \right)$$

7 $$B = \mathop \sum \limits_{t = 1}^{p} \left( {\sin \theta_{t} \times \overline{H}_{t} } \right)$$

8 $$CoA = arctan\left( {\frac{{\text{B}}}{{\text{A}}}} \right)$$

where “p” is the number of points of the running cycle (*p* = 200) and $\overline{{\text{H}} }$ is the motor primitive averaged over the 60 running cycles.

#### Width of motor primitives

We also compared the averaged motor primitives based on their full width at half maximum (FWHM), which describe their duration. It was calculated, after subtracting their minimum, as the number of points exceeding half of their maximum^25,26^. For each point of the running cycle (from 1 to 200), we also calculated the frequency at which motor primitives exceeded half of their maximum over the 60 running cycles, and the frequency at which they overlapped. Overlap occurred when at least two primitives exceeded half of their maximum at the same time^30,62^.

### Nonlinear metrics for the analysis of muscle synergies

#### Local complexity of motor primitives

To assess the local complexity of motor primitives (i.e. their “roughness” within each running cycle), we calculated their Higuchi’s fractal dimension^63^ (HFD). For each motor primitive H(t) = [H(1) H(2) … H(n)], we constructed k new times series as described in Eq. (9), where k ∈ ⟦2 ; k_max_⟧.

9 $${\text{H}}_{{\text{k}}}^{{t_{0} }} = [{\text{H}}\left( {t_{0} } \right){\text{H}}\left( {t_{0} + {\text{k}}} \right){\text{H}}\left( {t_{0} + 2{\text{k}}} \right) \ldots {\text{H}}\left( {t_{0} + \left\lfloor {\frac{{{\text{n}} - t_{0} }}{{\text{k}}}} \right\rfloor {\text{k}}} \right)$$

where ${t}_{0}$ ∈ ⟦1; k⟧ is the first sample at initial time and ⌊ ⌋ the integer part. Then, we calculated the non-Euclidean Higuchi length ${L}_{k}^{{t}_{0}}$ of each new time series ${H}_{k}^{{t}_{0}}$ as defined in Eq. (10).

10 $${\text{L}}_{{\text{k}}}^{{{\text{t}}_{0} }} = { }\frac{1}{{\text{k}}}{ }\left[ {{ }\frac{{{\text{n}} - 1}}{{\left\lfloor {\frac{{{\text{n}} - {\text{t}}_{0} }}{{\text{k}}}} \right\rfloor {\text{k}}}}\left( {\mathop \sum \limits_{{{\text{i}} = 1}}^{{\frac{{{\text{n}} - {\text{t}}_{0} }}{{\text{k}}}{\text{k}}}} \left| {{\text{H}}\left( {{\text{t}}_{0} + {\text{ik}}} \right) - {\text{H}}({\text{t}}_{0} + \left( {{\text{i}} - 1} \right){\text{k}}} \right|} \right)} \right]$$

The length of the motor primitive ${L}_{k}$ was defined as the average of the k sets lengths as shown in Eq. (11).

11 $${{\text{L}}}_{{\text{k}}}= \frac{1}{{\text{k}}} \sum_{{{\text{t}}}_{0}=1}^{{\text{k}}}{{\text{L}}}_{{\text{k}}}^{{{\text{t}}}_{0}}$$

If ${{\text{L}}}_{{\text{k}}}\propto {{\text{k}}}^{-{\text{HFD}}}$, then the curve is fractal with dimension HFD and this should lead the plot of ${{\text{log}}({\text{L}}}_{{\text{k}}})$ vs. ${\text{log}}\left(1/{\text{k}}\right)$ to fall on a straight line with slope HFD. The subsampling rate k was determined by repeating the previous operation for k = 1, 2, 3, … up to a maximum of k = 300, and then selecting the maximum value of k, k_max_, for which the curve of ${{\text{log}}({\text{L}}}_{{\text{k}}})$ vs. ${\text{log}}\left(1/{\text{k}}\right)$ was linear^31^. Our data led us to choose k_max_ = 10. HFD varies between 1 and 2, with increasing values corresponding to a more locally complex (or rough) signal, and the value 1.5 indicating a random Gaussian noise^63,64^.

#### Global complexity of motor primitives

To assess the global complexity of motor primitives (i.e. their irregularity across running cycles), we calculated their Hurst exponent (HE) following the rescaled range approach^65,66^. For each motor primitive H(t) = [H(1) H(2) … H(n)], we constructed a new time series of length ${\text{q}}$ (q ∈ ⟦1 ; n⟧), as described in Eq. (12).

12 $${{\text{H}}}_{{\text{q}}}= \begin{array}{cccc}[{\text{H}}(1)& {\text{H}}\left(2\right)& \dots & {\text{H}}\left({\text{q}}\right)\end{array}]$$

${H}_{q}$ was then centred by substracting its mean $\overline{{{\text{H}} }_{{\text{q}}}}$ and a series of cumulative values ${{\text{H}}}_{{\text{q}}}^{{\text{t}}}$ was established, as described in Eq. 13.

13 $${{\text{H}}}_{{\text{q}}}^{{\text{t}}}= \sum_{{\text{u}}=1}^{{\text{t}}}{{\text{H}}}_{{\text{q}}}({\text{u}})-\overline{{{\text{H}} }_{{\text{q}}}}$$

We determined the range ${{\text{R}}}_{{\text{q}}}$ of the time series ${{\text{H}}}_{{\text{q}}}^{{\text{t}}}$ (i.e. the difference between the maximum and minimum values) and its standard deviation ${{\text{S}}}_{{\text{q}}}$. We computed its rescaled range $({{\text{R}}}_{{\text{q}}}/{{\text{S}}}_{{\text{q}}})$. The value of q was determined by repeating the previous operations for q = n, n/2, n/4, n/8, … until a minimum of q = 2, and then selecting the value of q, q_inflex_, where an inflexion point occurred^31^. We finally calculated HE as the slope of the curve of ${\text{log}}\left({\text{q}}\right)$ *vs.* ${\text{log}}\left({{\text{R}}}_{{\text{q}}}/{{\text{S}}}_{{\text{q}}}\right)$ between q = 2 and q = q_inflex_. Our data led us to choose q_inflex_ = 200. HE can vary between 0 and 1. For 0.5 < HE < 1, the time series (i.e. motor primitive) is persistent, meaning that high values will probably be followed by other high values (and vice versa). For 0 < HE < 0.5, the time series is anti-persistent meaning that high values will probably be followed by low values (with frequent alternation between the two). HE = 0.5 indicates a completely random series without any persistence^66^.

### Functional classification of muscle synergies

All the above calculations were conducted only for the motor primitives relative to fundamental synergies. The recognition of fundamental synergies was carried out by k-means clustering to reduce possible operator bias in the classification. We first clustered the average motor primitives (over the 60 running cycles), for each condition separately. This classification operation was performed for a number of clusters ranging from 1 to 11 using the Hartigan Wong algorithm^67^. For each number of clusters, the classification was repeated 20 times with different initializations of the centroids. The solution that minimized the intra-class variance was retained. The number of clusters was determined by fitting the curve of intra-class variance *vs.* number of clusters with a simple linear regression model, and processing in the same way as for the determination of the number of synergies (until mean squared error was less than 10^–4^). The motor modules were then classified by imposing the same number of clusters as previously obtained for the motor primitives. The average FWHM and CoA of the motor primitives were then summed and normalized by the number of points (i.e. 200). This value was used as a score to compare the k-means clustering of motor primitives and motor modules. Consistent clusters identified fundamental synergies, while discordant clusters identified combined synergies (linear combination of other simpler synergies). Due to the lack of consensus in the literature on how to interpret combined synergies, they were excluded from the analysis.

### Statistics

For each variable, a linear mixed model (R package LmerTest^68^) was performed using restricted maximum likelihood estimation. Joint, Muscle, Synergy, Condition and Run were considered as fixed effects when appropriate, and preferred running speed as covariate. The intercepts for the participants and the slope per condition depending on the foot strike pattern were chosen as random effects. The significance of the random effects was tested. The best model (i.e. the number of fixed effects and interactions) was selected by likelihood ratio tests of model comparisons using a backward selection method. An Analysis of Variance (ANOVA) (degrees of freedom estimated with the Satterthwaite formula) was then performed on the selected model. This was followed by multiple comparisons with Tukey (for main effects) and Holm (for interaction effects) adjustment. If the normality of the residuals was violated (Shapiro–Wilk test), a permutation ANOVA of the linear mixed model was performed (R package lmPerm, number of permutations: 10,000). The effect size (ES) was calculated using Cohen’s d coefficient^69^ and assessed using the following thresholds: 0.2 to < 0.6, 0.6 to < 1.2 and 1.2 to < 2.0 for small, moderate and large effects, respectively^70^. In cases where motor primitives could not be compared using conventional linear metrics (CoA and FWHM) because they exhibited a bimodal profile, we used a two-way (Condition, Run) repeated measures ANOVA based on the one-dimensional Statistical Parametric Mapping analysis (SPM) (Python package spm1d)^35^. This was followed by multiple comparisons with Bonferroni adjustment. Finally, Spearman's correlation tests were used to quantify the correlations between the adjustments in motor primitives (in CoA, FWHM, HFD and HE) and those in the spatiotemporal parameters of the running pattern (in stance time, flight time and stride frequency). The magnitude of Spearman correlation coefficients was interpreted using the following thresholds: 0.1 to < 0.3, 0.3 to < 0.5 and > 0.5 for small, moderate and large, respectively^70^.

All the significance levels were set to α = 0.05 and the statistical analyses were performed using custom R and Python scripts (v3.9.7, 2021, Python Software Foundation, Wilmington, Delaware, USA). All results are presented in the text as estimated mean ± standard error.

### Supplementary Information

Supplementary Tables.

## Data Availability

The raw data and the codes developed for their analysis are available to any researcher upon request to the corresponding author: Camille Fazzari (camille.fazzari@univ-amu.fr).
