# Supplementary material for: Muscle synergies inherent in simulated hypogravity running reveal flexible but not unconstrained locomotor control
Source: Sci Rep. 2024 Feb 1;14:2707. doi: 10.1038/s41598-023-50076-6 (PMC10834966; doi:10.1038/s41598-023-50076-6)
Supplement: Supplementary file 1 — Supplementary Tables. [file 41598_2023_50076_MOESM1_ESM.docx]

**Muscle synergies inherent in simulated hypogravity running reveal**

**flexible but not unconstrained locomotor control**

Camille Fazzari, Robin Macchi, Yoko Kunimasa, Camélia Ressam, Rémy Casanova, Pascale Chavet, Caroline Nicol

**Supplementary Table 1**. Spatiotemporal adjustments from HYPO to RLD and INIT to RLD. Significant adjustments (in gray) are shown as estimate mean ± standard error (effect size), non-significant as ns. P-values were obtained after multiple comparisons with Tukey’s adjustment.

|  |  | **From HYPO to RLD** | **From INIT to RLD** |
| --- | --- | --- | --- |
| **Stance time** | Δ  p-value | +12 ± 5% (1.3)  < 0.001 | ns  0.67 |
| **Flight time** | Δ  p-value | -20 ± 5% (2.0)  < 0.001 | ns  0.60 |
| **Stride frequency** | Δ  p-value | +23 ± 4% (2.7)  < 0.001 | -3 ± 27% (0.3)  0.001 |

**Supplementary Table 2.** Adjustment in the number of muscle synergies required to reconstruct the original sEMG signals and in the reconstruction quality from HYPO to RLD and INIT to RLD. Significant adjustments (in gray) are shown as estimate mean ± standard error (effect size), non-significant as ns. P-values were obtained after multiple comparisons with Tukey’s adjustment.

|  |  | **From HYPO to RLD** | **From INIT to RLD** |
| --- | --- | --- | --- |
| **Number of synergies** | Δ  p-value | ns  0.70 | ns  0.21 |
| **Reconstruction quality** | Δ  p-value | +2 ± 17% (0.8)  < 0.001 | ns  0.70 |

**Supplementary Table 3**. Number of participants (out of 38) presenting each muscle synergy.

|  | **Braking** | **Push-off** | **Early flight** | **S4** |
| --- | --- | --- | --- | --- |
| **INIT** | 38 | 38 | 33 | 37 |
| **HYPO** | 37 | 36 | 35 | 24 |
| **RLD** | 38 | 38 | 34 | 37 |

**Supplementary Table 4**. Motor modules adjustments from HYPO to RLD and INIT to RLD. Significant adjustments (in gray) are shown as estimate mean ± standard error (effect size), non-significant as ns. P-values were obtained after multiple comparisons with Holm’s adjustment.

|  |  | **From HYPO to RLD** | | | | **From INIT to RLD** | | | |
| --- | --- | --- | --- | --- | --- | --- | --- | --- | --- |
|  |  | **Braking** | **Push-off** | **Early flight** | **S4** | **Braking** | **Push-off** | **Early flight** | **S4** |
| **Muscle contributions** | | | | | | | | | |
| **TA** | Δ  p-value | ns  1 | ns  1 | ns  1 | +130 ± 23% (0.7)  < 0.01 | ns  1 | ns  1 | ns  1 | ns  1 |
| **GaM, GaL** | Δ  p-value | ns  1 | ns  1 | ns  1 | ns  1 | ns  1 | ns  1 | ns  1 | ns  1 |
| **SOL** | Δ  p-value | ns  1 | ns  0.13 | ns  1 | ns  1 | ns  1 | ns  1 | ns  1 | ns  1 |
| **PL** | Δ  p-value | ns  1 | +19 ± 26% (0.6)  < 0.05 | ns  1 | ns  1 | ns  1 | ns  1 | ns  1 | ns  1 |
| **VM, VL** | Δ  p-value | ns  1 | ns  1 | ns  1 | ns  1 | ns  1 | ns  1 | ns  1 | ns  1 |
| **RF** | Δ  p-value | ns  1 | ns  1 | +71 ± 25% (0.5)  < 0.01 | ns  1 | ns  1 | ns  1 | ns  1 | ns  1 |
| **STSM** | Δ  p-value | ns  1 | ns  0.33 | -71 ± 16% (0.8)  < 0.001 | ns  1 | ns  1 | ns  1 | ns  1 | ns  1 |
| **BF** | Δ  p-value | ns  1 | ns  1 | -56 ± 22% (0.8)  < 0.001 | ns  1 | ns  1 | ns  1 | ns  1 | ns  1 |
| **GM** | Δ  p-value | -17 ± 24% (0.4)  < 0.01 | ns  1 | ns  0.35 | ns  1 | ns  1 | ns  1 | ns  1 | ns  1 |
| **Co-contribution index** | | | | | | | | | |
| **Hip** | Δ  p-value | ns  1 | ns  1 | +39 ± 25% (0.6)  < 0.01 | ns  0.32 | ns  1 | ns  0.46 | ns  1 | ns  1 |
| **Knee** | Δ  p-value | ns  1 | ns  0.36 | +63 ± 15% (0.8)  < 0.001 | ns  1 | ns  1 | ns  0.46 | ns  0.14 | ns  1 |
| **Ankle** | Δ  p-value | ns  1 | ns  1 | ns  1 | +70 ± 16% (0.8)  < 0.001 | ns  1 | ns  1 | ns  1 | ns  1 |

**Supplementary Table 5**. Motor primitive adjustments from HYPO to RLD and INIT to RLD. Significant adjustments (in gray) are shown as estimate mean ± standard error (effect size), non-significant as ns. P-values were obtained after multiple comparisons with Holm’s adjustment.

|  |  | **From HYPO to RLD** | | | | **From INIT to RLD** | | | |
| --- | --- | --- | --- | --- | --- | --- | --- | --- | --- |
|  |  | **Braking** | **Push-off** | **Early flight** | **S4** | **Braking** | **Push-off** | **Early flight** | **S4** |
| **CoA** | Δ  p-value | ns  0.41 | -15 ± 25% (1.1)  < 0.001 | -6 ± 19% (0.5)  < 0.001 | Excluded from the analysis | ns  1 | ns  1 | ns  0.1 | Excluded from the analysis |
| **FWHM** | Δ  p-value | ns  0.06 | -11 ± 37% (0.7)  < 0.05 | ns  0.43 | Excluded from the analysis | ns  1 | ns  1 | ns  0.06 | Excluded from the analysis |
| **HFD** | Δ  p-value | -2 ± 19% (0.9)  < 0.001 | ns  1 | -3 ± 13% (0.8)  < 0.001 | -5 ± 10% (1.4)  < 0.001 | ns  1 | ns  1 | ns  1 | ns  1 |
| **HE** | Δ  p-value | -20 ± 22% (0.9)  < 0.001 | -20 ± 20% (0.9)  < 0.001 | -13 ± 24% (0.5)  < 0.001 | -21 ± 14% (1.4)  < 0.001 | ns  1 | ns  1 | ns  1 | ns  0.28 |

**Supplementary Table 6**. Spearman’s rank correlations between the relative adjustments in the timing (CoA) and duration (FWHM) of motor primitives and in the spatiotemporal parameters of running from INIT to HYPO and HYPO to RLD.

|  |  | **CoA** | | | | **FWHM** | | | |
| --- | --- | --- | --- | --- | --- | --- | --- | --- | --- |
|  |  | **Braking** | **Push-off** | **Early flight** | **S4** | **Braking** | **Push-off** | **Early flight** | **S4** |
| **From INIT to HYPO** | | | | | | | | | |
| **Stance time** | ρ  p-value | 0.21  0.21 | -0.13  0.44 | 0.28  0.14 | Excluded from the CoA analysis | 0.15  0.24 | -0.41  < 0.05 | -0.33  0.08 | Excluded from the FWHM analysis |
| **Flight time** | ρ  p-value | 0.26  0.12 | 0.35  < 0.05 | 0.31  0.09 |  | 0.41  < 0.05 | 0.03  0.87 | -0.15  0.44 |  |
| **Stride frequency** | ρ  p-value | -0.14  0.40 | -0.11  0.52 | -0.29  0.12 |  | -0.31  0.06 | -0.01  0.95 | -0.70  0.07 |  |
| **From HYPO to RLD** | | | | | | | | | |
| **Stance time** | ρ  p-value | 0.27  0.11 | 0.03  0.85 | 0.44  < 0.05 | Excluded from the CoA analysis | 0.28  0.09 | -0.29  0.09 | -0.50  < 0.01 | Excluded from the FWHM analysis |
| **Flight time** | ρ  p-value | 0.22  0.21 | 0.32  0.06 | 0.64  < 0.001 |  | 0.21  0.22 | -0.21  0.24 | -0.18  0.32 |  |
| **Stride frequency** | ρ  p-value | -0.14  0.41 | -0.18  0.28 | -0.52  < 0.01 |  | -0.41  < 0.05 | 0.18  0.29 | 0.23  0.20 |  |

**Supplementary Table 7**. Spearman’s rank correlations between the relative adjustments in the local (HFD) and global (HE) complexity of motor primitives and in the spatiotemporal parameters of running from INIT to HYPO and HYPO to RLD.

|  |  | **HFD** | | | | **HE** | | | |
| --- | --- | --- | --- | --- | --- | --- | --- | --- | --- |
|  |  | **Braking** | **Push-off** | **Early flight** | **S4** | **Braking** | **Push-off** | **Early flight** | **S4** |
| **From INIT to HYPO** | | | | | | | | | |
| **Stance time** | ρ  p-value | 0.07  0.66 | 0.51  < 0.01 | -0.14  0.44 | 0.20  0.36 | -0.14  0.39 | -0.05  0.79 | -0.27  0.15 | -0.13  0.54 |
| **Flight time** | ρ  p-value | 0.20  0.23 | 0.13  0.44 | 0.30  0.11 | 0.25  0.26 | 0.06  0.73 | -0.06  0.72 | -0.07  0.69 | 0.35  0.10 |
| **Stride frequency** | ρ  p-value | -0.33  < 0.05 | -0.18  0.30 | -0.32  0.08 | -0.44  < 0.05 | -0.18  0.28 | -0.13  0.45 | -0.07  0.72 | -0.52  < 0.05 |
| **From HYPO to RLD** | | | | | | | | | |
| **Stance time** | ρ  p-value | 0.12  0.47 | -0.62  < 0.001 | -0.19  0.29 | 0.32  0.12 | -0.14  0.39 | -0.05  0.79 | -0.27  0.15 | -0.13  0.53 |
| **Flight time** | ρ  p-value | -0.04  0.80 | 0.18  0.30 | -0.08  0.68 | -0.19  0.38 | -0.14  0.39 | -0.04  0.79 | -0.27  0.15 | -0.13  0.54 |
| **Stride frequency** | ρ  p-value | -0.31  0.06 | -0.30  0.08 | -0.04  0.82 | -0.27  0.19 | 0.05  0.79 | -0.02  0.90 | -0.13  0.49 | -0.46  < 0.05 |
